# Identification of acetylated diether lipids in halophilic Archaea

**DOI:** 10.1002/mbo3.1299

**Published:** 2022-06-09

**Authors:** Cosimo Kropp, Julius Lipp, Anna Lena Schmidt, Christina Seisenberger, Mona Linde, Kai‐Uwe Hinrichs, Patrick Babinger

**Affiliations:** ^1^ Institute of Biophysics and Physical Biochemistry, Regensburg Center for Biochemistry University of Regensburg Regensburg Germany; ^2^ MARUM Center for Marine Environmental Sciences University of Bremen Bremen Germany; ^3^ Present address: Roche Diagnostics GmbH Penzberg Germany; ^4^ Present address: Boehringer Ingelheim Pharma GmbH & Co. KG. Biberach an der Riß Germany

**Keywords:** acetyltransferase, archaeol, ether lipids, Halobacteria, mass spectrometry

## Abstract

As a hallmark of Archaea, their cell membranes are comprised of ether lipids. However, Archaea‐type ether lipids have recently been identified in Bacteria as well, with a somewhat different composition: In Bacillales, *sn*‐glycerol 1‐phosphate is etherified with one C35 isoprenoid chain, which is longer than the typical C20 chain in Archaea, and instead of a second isoprenoid chain, the product heptaprenylglyceryl phosphate becomes dephosphorylated and afterward diacetylated by the *O*‐acetyltransferase YvoF. Interestingly, database searches have revealed YvoF homologs in Halobacteria (Archaea), too. Here, we demonstrate that YvoF from *Haloferax volcanii* can acetylate geranylgeranylglycerol in vitro. Additionally, we present the first‐time identification of acetylated diether lipids in *H. volcanii* and *Halobacterium salinarum* by mass spectrometry. A variety of different acetylated lipids, namely acetylated archaeol, and acetylated archaetidylglycerol, were found, suggesting that halobacterial YvoF has a broad substrate range. We suppose that the acetyl group might serve to modify the polarity of the lipid headgroup, with still unknown biological effects.

## Graphical abstract

Archaea‐type ether lipids have been found recently in Bacillales. They are monoether lipids with a C35 isoprenoid chain and are diacetylated by the *O*‐acetyltransferase YvoF. Phylogenetic studies implied that YvoF homologs also occur in Halobacteria (Archaea). Here we show that YvoF from *Haloferax volcanii* has ether‐lipid‐specific acetyltransferase activity in vitro. Furthermore, we provide a mass spectrometric identification of acetylated archaeol and acetylated archaetidylglycerol in *H. volcanii* and *Halobacterium salinarum* for the first time.

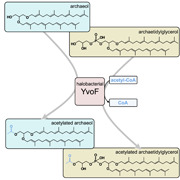

## 1 INTRODUCTION

**Figure 1:**
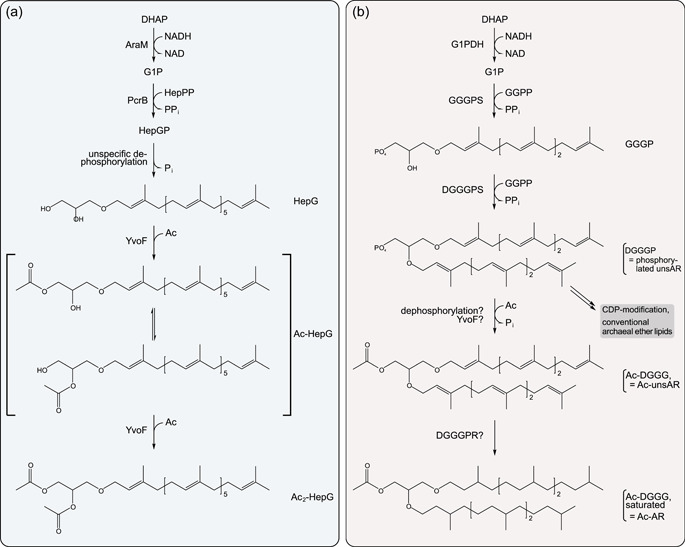
Biosynthesis of ether lipids in Bacteria and Archaea. (a) Biosynthetic pathway as discovered in *Bacillus subtilis.* The figure is adapted from Linde et al. (2016). The acetyl moiety in Ac‐HepG can putatively swap between the hydroxyl groups by acyl migration. (b) Postulated biosynthetic pathway of acetylated ether lipids in Halobacteria. Ac, acetyl group; AG, archaetidylglycerol (corresponding to 2,3‐diphytanyl‐sn‐glycerol‐1‐phospho‐3'‐sn‐glycerol); AR, archaeol (corresponding to 2,3‐diphytanyl‐sn‐glycerol); AraM, G1PDH of B. subtilis; CDP, cytidine diphosphate; DGGGP, digeranylgeranylglyceryl phosphate (corresponding to phosphorylated unsaturated archaeol (unsAR)); DGGGPR, digeranylgeranylglyceryl phosphate reductase; DGGGPS, digeranylgeranylglyceryl phosphate synthase; DHAP, dihydroxyacetone phosphate; G1PDH, glycerol 1‐phosphate dehydrogenase; GGG, geranylgeranylglycerol; GGGP, geranylgeranylglyceryl phosphate; GGGPS, geranylgeranylglyceryl phosphate synthase; GGPP, geranylgeranyl pyrophosphate; HepGP, heptaprenylglyceryl phosphate; HepPP, heptaprenyl pyrophosphate; NAD, nicotinamide adenine dinucleotide; Pi, phosphate; PPi, diphosphate; PcrB, GGGPS homolog of B. subtilis; uns, unsaturated.

Membrane lipids of Archaea and Bacteria differ in some respects. In Archaea, their backbone consists of *sn*‐glycerol 1‐phosphate (G1P), to which isoprenoids, typically 20 C‐atoms in length (C20), are linked via an ether bond (Jain et al., 2014). In Bacteria, *sn*‐glycerol 3‐phosphate (G3P) is esterified with fatty acids. The separation of Archaea and Bacteria during evolution has been postulated to be driven by the emergence of the enzymes synthesizing the membrane lipids, which has been summarized under the term “lipid divide” (Boucher, 2007; Boucher et al., 2004; Glansdorff et al., 2008; Koga, 2011; Koga & Morii, 2007; Lombard et al., 2012; Payandeh & Pai, 2007; Pereto et al., 2004; Villanueva et al., 2017). For a long time, lipids consisting of isoprenoids ether linked to G1P were considered exclusive for the archaeal domain, until the recent identification of Archaea‐type ether lipids in gram‐positive *Bacillus subtilis* (Guldan et al., 2008, 2011). Correspondingly, homologs to the enzyme that links the first isoprenoid chain to G1P, geranylgeranylglyceryl phosphate synthase (GGGPS), have been found in Bacillales and also in gram‐negative Bacteroidetes (Peterhoff et al., 2014). However, the ether lipids found in *B. subtilis* are quite different compared to those in Archaea. While in Archaea, commonly a second C20 isoprenoid chain is attached to the glycerol by digeranylgeranylglyceryl phosphate synthase (DGGGPS), ether lipids in *B. subtilis* possess one C35 chain (Guldan et al., 2011; Peterhoff et al., 2014). The glycerol moiety furthermore becomes dephosphorylated and acetylated, which is catalyzed by the acetyl‐CoA‐dependent *O*‐acetyltransferase YvoF, resulting in mono‐ or diacetylated derivatives (Figure 1a) (Guldan et al., 2011; Linde et al., 2016). Their biological function in Bacteria is still an enigma.

**Figure A1:**
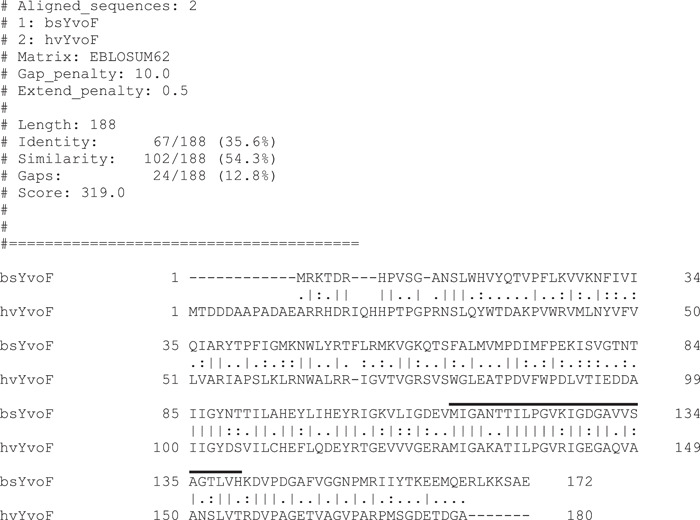
Pairwise sequence alignment of YvoF from *Bacillus subtilis* (bsYvoF) and *Haloferax volcanii YvoF* (hvYvoF). The alignment was created using the Needleman–Wunsch algorithm. The numbers of identical and similar amino acids are given in the header. The acetyl‐CoA binding site in bsYvoF (residues 114‐140 according to GenBank entry KIX83267.1) is marked with a black bar.

Database searches have indicated that YvoF homologs exist in many Halobacteria as well (Linde et al., 2016), which was quite surprising as acetylated ether lipids have not been known to exist in Archaea so far. Halobacterial YvoF sequences share an overall identity of about 50% among each other, and about 35%–45% with bacterial YvoF representatives; however, at the acetyl‐CoA binding site, the similarity is significantly higher (Figure A1). Since confirmation of the activity of YvoF from Halobacteria as well as identification of acetylated ether lipids within this phylogenetic clade has still been missing, we purified YvoF from *Haloferax volcanii YvoF* (hvYvoF) and verified acetyltransferase activity with bacterial C20 monoether lipids as substrate. We subsequently performed mass spectrometry (MS) to tackle the identification of acetylated ether lipids in extracts from *H. volcanii* and *Halobacterium salinarum*. Our results let us postulate a pathway for the synthesis of acetylated diether lipids in Halobacteria (Figure 1b). We also discovered further acetylated phospholipids in the two halobacterial strains and discuss their occurrence in the context of YvoF.

## 2 MATERIALS AND METHODS

### 2.1 Halobacterial strains and culture media

For lipid analysis, *H. volcanii* H1424 (Stroud et al., 2012) and *H. salinarum* ATCC 700922 (Ng et al., 2000) were used. hvYvoF was produced in *H. volcanii* H1424. Both strains were obtained from Sébastien Ferreira‐Cerca, University of Regensburg.

*H. volcanii* was grown in a salt‐rich hv‐YPC medium at 42°C. For nonplasmid‐harboring strains, the medium was supplemented with 40 µg ml^−1^ thymidine and for plasmid‐harboring strains with 75 μg ml^−1^ kanamycin (Allers et al., 2004; Guy et al., 2006). *H. salinarum* was grown in the medium for extreme halophiles at 42°C (Cline et al., 1989).

### 2.2 Cloning

hv*Y* *voF* was amplified by a polymerase chain reaction from *H. volcanii* H1424 genomic DNA. The primers used for amplification and cloning are given in Table A1. The produced fragments were cloned via NdeI/XhoI into the pTA1228 expression vector (Allers et al., 2010) providing a C‐terminal hexahistidine (His)_6_ tag.

### 2.3 Transformation procedures

Transformation of *Escherichia coli* for cloning was performed using a standard protocol for chemically competent cells (Sambrook et al., 1989). Production of competent cells of *H. volcanii* and polyethylene‐mediated transformation was performed as described by Cline et al. (1989). The used solutions and media were produced accordingly.

### 2.4 Production and purification of recombinant hvYvoF

Heterologous gene expression was performed in *H. volcanii* H1424 (Allers et al., 2010). To this end, 81.7 mg l‐tryptophan were dissolved in 360 mL hv‐YPC medium (1.11 mM final concentration) by shaking at 180 rpm and 42°C in 3 L flasks. The medium was inoculated with an overnight culture (40 ml from the preculture diluted to 1 OD_600_) of transformed *H. volcanii* cells to 0.1 OD_600_ and shaking was continued until 0.5 OD_600_ was reached. Gene expression was induced a second time by adding 2 mM l‐tryptophan (final concentration) in 18% saltwater and incubation was continued for 16 h. Cells were harvested by centrifugation, resuspended in 40 ml 50 mM Tris, pH 8, 600 mM NaCl, 10 mM imidazole, and disrupted by sonication until the suspension was nonturbid anymore. Cell debris was removed by centrifugation. The His‐tagged protein was purified from the clarified cell extract by immobilized metal ion affinity chromatography (IMAC). An ÄKTApurifier system with a HisTrap FF Crude column (5 ml; Cytiva) was used, and a linear gradient of imidazole (10–500 mM) in 50 mM Tris, pH 8, 600 mM NaCl was applied to elute the protein. To remove interfering salts and imidazole, the protein was subjected to dialysis against 50 mM Tris, pH 8.0, and 600 mM NaCl. Protein concentrations were determined by absorbance spectroscopy using a Jasco V650 spectrophotometer. The molar extinction coefficients ε_280_ and the molecular weight were calculated from the amino acid sequence using ProtParam (Gasteiger et al., 2005). Purified protein was dropped into liquid nitrogen and stored at −80°C. UV‐Vis spectra for analysis of bound ligands were recorded using 10 µM protein (final concentration) in 50 mM Tris, pH 8, 600 mM NaCl (200–600 nm; response time 0.96 s; scan rate 40 nm min^−1^; bandwidth 2 nm).

### 2.5 Nano differential scanning fluorimetry (nanoDSF)

hvYvoF was heated at a final subunit concentration of 20 µM in 50 mM Tris, pH 8.0 buffer from 20°C to 95°C (1 K min^‐1^), in a Prometheus NT.48 instrument (NanoTemper Technologies GmbH; access provided by 2bind; 10% excitation power at 280 nm). The change in the ratio of the fluorescence signal at 350–330 nm with raising temperature was monitored and the fluorescence transitions were evaluated using the program supplied by the manufacturer. The apparent melting point temperature (*T*_M_) of the irreversible unfolding transition was determined as an operational measure of thermal protein stability. Experiments were done in duplicates, which overlapped perfectly.

### 2.6 ^14^C‐GGG activity assay

^14^C‐GGGP was synthesized as described previously (Linde et al., 2016) in 50 mM Tris, pH 8.0, 10 mM MgCl_2_, 0.2% Tween80, and dephosphorylated to ^14^C‐GGG by adding 1 U of calf intestinal phosphatase (CIP) and further incubation at 40°C for 1 h. To test the activity of the purified hvYvoF enzyme, 1 µM hvYvoF, 0.25 mM acetyl‐CoA (Sigma), and varying concentrations of NaCl were added to the synthesized ^14^C‐GGG (2.5 µM; 37.5 nCi) and incubated at 40°C. To visualize time‐dependent activity, the reaction was stopped at different time points by the addition of chloroform. The products were extracted according to the method of Bligh and Dyer (Bligh & Dyer, 1959) as modified by Kates (1986). The extract was analyzed on Silica 60 plates developed in ethyl acetate:hexane 1:1 (v:v) and visualized with a phosphorimager system (PerkinElmer Life Sciences).

### 2.7 Assay for substrate range of hvYvoF

GGG with and without the ^14^C label was synthesized as described above, but the incubation time for dephosphorylation was only 30 min. The buffer for all samples was 50 mM Tris pH 7.5, 100 mM NaCl, 10 mM MgCl_2_, 0.2% Tween80. A total of 20 µM GGG, or 500 µM monoacylglycerol (1‐decanoyl‐rac‐glycerol) were incubated with 1 µM hvYvoF and 1 mM unlabeled or a mixture of unlabeled and [1‐^14^C]‐labeled acetyl‐CoA (40 nCi; American Radiolabeled Chemicals) for 2 h at 40°C. All samples were extracted and analyzed as described above.

### 2.8 Production of total lipid extract (TLE)

*H. volcanii* and *H. salinarum* were grown at 42°C. Cells were harvested by centrifugation and stored at −80°C. Lipid extraction was performed in a one‐step procedure based on Wörmer et al. (2015). A hundred milligrams of each strain were washed into a 50 ml teflon vessel using 20 ml of Bligh and Dyer extraction (BDE) mix A containing MeOH:dichloromethane (DCM):PO_4_ buffer in a ratio of 2:1:0.8 (v:v:v). The vessel was sealed, mixed thoroughly by shaking, and subjected to sonication in an ultrasonic bath for 10 min. Afterward, the mixture was transferred into a 100 ml separating funnel and 3 ml DCM and 6.3 ml H_2_O were added. After intensive shaking, the separating funnel was vented and left for phase separation. Upon completion of the separation, the organic bottom layer was transferred into a 40 ml evaporation vessel. The remaining aqueous phase in the separating funnel was washed with 5 ml DCM and left for phase separation. The organic phase was carefully drawn off and added to the evaporation vessel. The washing step was repeated three times. Finally, the aqueous phase was discarded and the separating funnel was washed with DCM and MeOH. The organic phase was now transferred to the funnel and washed three times with 5 ml purified H_2_O in the same way as described for the previous washing procedure. After the third repetition, the organic phase, now representing the TLE, was put into an evaporator (TurboVap, Biotage) in a water bath set to 40°C and the solvent was evaporated to dryness under a nitrogen stream. The sample was re‐dissolved in a solvent containing DCM:MeOH in a ratio of 5:1, transferred into 4 ml vials, and evaporated under N_2_ flow at 40°C (Merck Supelco). The TLE was stored at −20°C until further use.

### 2.9 RP‐UHPLC‐ESI‐MS

The TLE was dissolved in 2 ml of DCM:MeOH (5:1, v:v) and 400 µl (20% of the TLE) was transferred into a new vial. C46‐GTGT standard (Huguet et al., 2006) was added to a final amount of 6 ng. The mix was evaporated under a gentle N_2_ flow and re‐dissolved in 20 µl MeOH:DCM (9:1, v:v). From that, 10 µl were injected (equal to 10% of the TLE and 3 ng standard) for analysis in RP‐UHPLC‐ESI‐MS.

For analysis of the intact polar lipids, the prepared TLE dilution was separated by ultrahigh performance liquid chromatography (UHPLC) on a Dionex ultimate 3000RS system (Thermo Fisher Scientific) coupled to a Bruker quadrupole time‐of‐flight mass spectrometer (QTOF‐MS; Bruker Daltonics). At the end of each analysis, a tune mix solution was injected via a 20 µl loop to ensure mass calibration for each analysis, and additionally, a lock mass calibration was applied to correct each mass spectrum. The resulting mass accuracy was better than 3 ppm. To separate the compounds mainly by alkyl chain hydrophobicity, the system was operated using an Acquity BEH C18 column (1.7 µm, 2.1 × 150 mm) (Waters). The flow rate was set to 0.4 ml min^−1^, column temperature at 65°C, and a gradient program using reversed‐phase electrospray ionization (RP‐ESI) mobile phase A and B buffer was programmed according to literature (Wörmer et al., 2013, 2015). Mobile phase A was MeOH:H_2_O 85:15 (v:v), 0.04% formic acid, 0,1% NH_4_OH, mobile phase B isopropanol:MeOH 50:50 (v:v), 0.04% formic acid, 0.1% NH_4_OH. Output data were analyzed using software supplied by the manufacturer (DataAnalysis 5.0; Bruker Daltonics).

## 3 RESULTS AND DISCUSSION

### 3.1 Activity of *H. volcanii* YvoF in vitro

**Figure A2:**
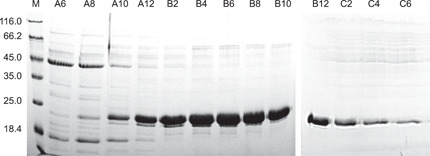
Purification of hvYvoF by IMAC. Fractions with a volume of 5 ml were collected during chromatography, and 30 µl of every second fraction was analyzed on a 13.5% sodium dodecylsulfate‐polyacrylamide gel electrophoresis gel. Fractions A12‐C6 were pooled and used for further experiments. Size marker (M), MW in kDa. The calculated molecular mass of hvYvoF is 20.5 kDa. The samples were run on two gels, as indicated by the boundary line. IMAC, immobilized metal chelate ion affinity chromatography; hvYvoF, *Haloferax volcanii* *YvoF*; SDS‐PAGE, sodium dodecylsulfate‐polyacrylamide gel electrophoresis.

**Figure A3:**
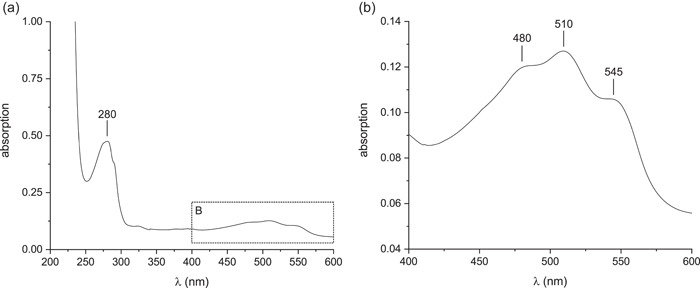
The absorption spectrum of hvYvoF. The spectrum was recorded using 10 µM hvYvoF in 50 mM Tris‐HCl, pH 8.0, 600 mM NaCl from (a) 200 to 600 nm and from (b) 400 to 600 nm at 25°C (*d* = 0.4 cm). The box in (a) marks the section shown in (b). The wavelengths of the absorption maxima are indicated. hvYvoF, *Haloferax volcanii* *YvoF*.

**Figure A4:**
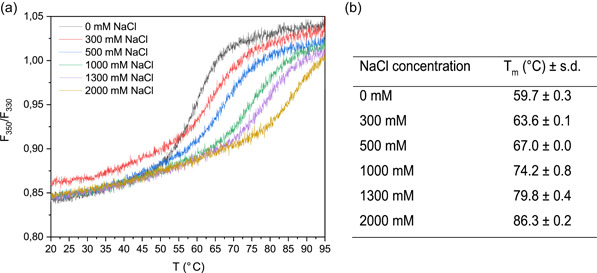
Thermal stability of hvYvoF, measured by nanoDSF. (a) The change in the ratio of fluorescence emission of 20 µM protein at 350 and 330 nm was monitored from 20°C to 95°C at a scan rate of 1 K min^‐^^1^. The protein was dissolved in 50 mM Tris‐HCl pH 8.0 and supplemented with varying NaCl concentrations (as indicated in the legend) (b) Apparent melting temperatures (*T*_m_) were derived as the inflection points of the curves from duplicates; the standard deviation is given. hvYvoF, *Haloferax volcanii* *YvoF*.

Because of the difficulties to express genes from halophilic species in *E. coli* due to the demand for high salt conditions, we produced YvoF from *Haloferax volcanii YvoF* (hvYvoF) in an *H. volcanii* expression system (Allers et al., 2010). To this end, we amplified the gene from genomic DNA and cloned it into the pTA1228 expression vector. After the transformation of chemically competent *H. volcanii* cells, expression was induced. Subsequent purification of hvYvoF was performed by IMAC and controlled by sodium dodecylsulfate‐polyacrylamide gel electrophoresis (SDS‐PAGE) (Figure A2). The purified protein solution was colored pink, indicating that carotenoids such as lycopene and bacterioruberin, which are abundant in *H. volcanii* cells (Ronnekleiv, 1995), were copurified with hvYvoF. We assume that due to their isoprenoid nature, carotenoids bind to hvYvoF in the binding pocket of its isoprenoid substrate. Following this assumption, the UV‐Vis spectrum of purified hvYvoF in the range of 450–550 nm is characteristic of bacterioruberin (Figure A3) (Dummer et al., 2011). In thermal denaturation experiments using differential scanning fluorimetry (nanoDSF), hvYvoF showed cooperative transition curves (Figure A4A). As expected for a protein from a halophilic organism, hvYvoF was significantly stabilized by increasing salt concentrations. The apparent midpoint temperature of the unfolding transition raised by about 27 K when NaCl was increased from 0 to 2000 mM (Figure A4B).

**Figure 2:**
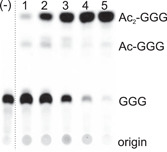
Test for GGG‐specific acetyltransferase activity of hvYvoF. hvYvoF at a concentration of 1 µM was incubated with 0.25 mM acetyl‐CoA and ^14^C‐GGG in 50 mM Tris‐HCl, pH 8.0, 10 mM MgCl_2_, 0.2% Tween80, 1300 mM NaCl for different periods of time at 40°C: (1) 0.1, (2) 2, (3) 10, (4) 30, (5) 120 min. A negative control (−) was performed without added hvYvoF. Reaction products were extracted, separated by TLC, and visualized by autoradiography. The origin of chromatography as well as spots of GGG, single acetylated Ac‐GGG, and double acetylated Ac_2_‐GGG are marked. The figure is a composition of sections from Figure A5, as indicated by the boundary line. GGG, geranylgeranylglycerol; hvYvoF, *Haloferax volcanii YvoF.*

To verify acetyltransferase activity, we performed an activity assay with a ^14^C‐labeled substrate as described previously (Linde et al., 2016). In brief, we synthesized radiolabeled geranylgeranylglyceryl phosphate (^14^C‐GGGP), which is the Archaea‐type ether lipid as identified in *B. subtilis*, but with a C20 instead of a C35 isoprenoid. ^14^C‐GGGP was incubated with CIP, generating geranylgeranylglycerol (^14^C‐GGG), and hvYvoF as well as acetyl‐CoA as donors for the acetyl group. The reaction was stopped by chloroform addition at different points in time. Afterward, the products were extracted, separated by thin‐layer chromatography (TLC), and visualized with a phosphoimager system (Figure 2).

**Figure A5:**
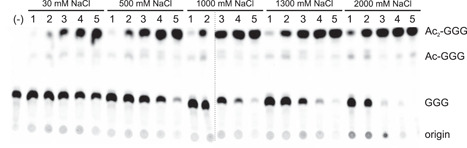
Test of salt‐dependent activity of hvYvoF. 1 µM hvYvoF was incubated with 0.25 mM acetyl‐CoA and ^14^C‐GGG in 50 mM Tris‐HCl, pH 8.0, 10 mM MgCl_2_, 0.2% Tween80, and 0‐2000 mM NaCl at 40°C for different periods of time at 40°C: (1) 0.1, (2) 2, (3) 10, (4) 30, (5) 120 min. The concentration of NaCl in the reaction buffer was varied as indicated. A negative control (−) was performed without added hvYvoF. Reaction products were extracted, separated by TLC, and visualized by autoradiography. The origin of chromatography as well as spots of GGG, single acetylated Ac‐GGG, and double acetylated Ac_2_‐GGG are marked. Chromatography was run on two separate TLC plates, as indicated by the boundary line. GGG, geranylgeranylglycerol; hvYvoF, *Haloferax volcanii* *YvoF*; TLC, thin‐layer chromatography.

TLC analysis showed that GGG was gradually diacetylated (to Ac_2_‐GGG) by hvYvoF with increasing incubation time. The monoacetylated intermediate Ac‐GGG was scarcely detected. Diacetylation such as previously observed in *B. subtilis* (Figure 1a) is possible in this case, as two hydroxyl groups are available for acetylation when using GGG as substrate. In Archaea, however, the second hydroxyl group is normally occupied by another isoprenoid chain, which is also seen later in MS experiments with in vivo samples. Because hvYvoF is a protein from a halophilic organism, we tested the dependence of activity upon increasing salt concentration in the assay (Figure A5). As expected, the activity was significantly increased in the presence of salt, which became most clearly visible in the faster decrease of the substrate GGG over the incubation time.

### 3.2 Identification of acetylated diether lipids in Halobacteria

**Figure 3:**
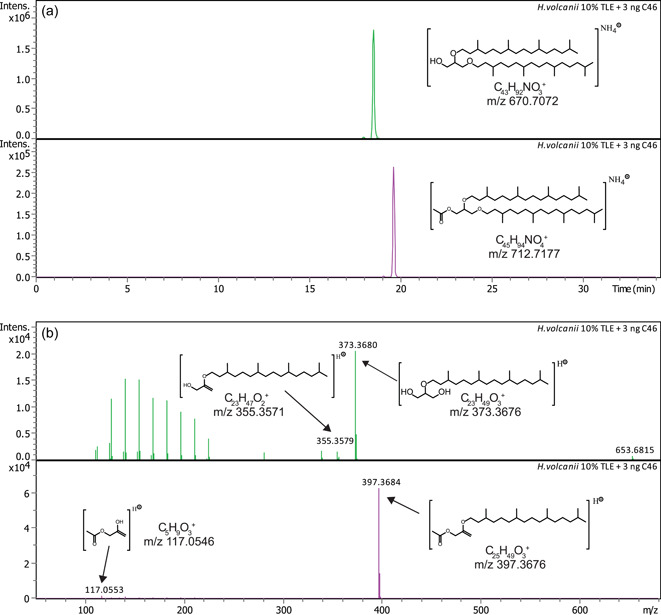
RP‐UHPLC‐ESI‐MS analysis of a *Haloferax volcanii* total lipid extract. (a) Extracted ion chromatograms of *m*/*z* 670.7072 ± 0.01 representing archaeol (AR, upper panel) and of *m*/*z* 712.7177 ± 0.01 representing acetylated archaeol (Ac‐AR, bottom panel). (b) Fragment mass spectra of AR (upper panel) and Ac‐AR (lower panel). Tentative structures and elemental formulas of diagnostic peaks are shown. C46 refers to the added standard (cf. Experimental Procedures). RP‐UHPLC‐ESI‐MS, reversed‐phase ultrahigh‐performance liquid chromatography coupled with electrospray ionization mass spectrometry.

**Figure A6:**
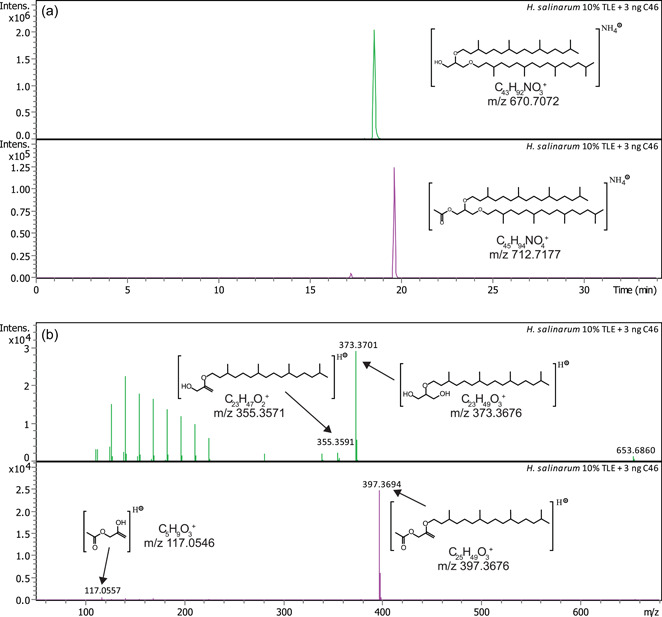
RP‐UHPLC‐ESI‐MS analysis of a *Halobacterium salinarum* total lipid extract. (a) Extracted ion chromatograms of *m*/*z* 670.7072 ± 0.01 representing archaeol (AR; upper panel) and of *m*/*z* 712.7177 ± 0.01 representing acetylated archaeol (Ac‐AR; bottom panel). (b) Fragment mass spectra of archaeol (upper panel) and acetylated archaeol (lower panel). Tentative structures and elemental formulas of diagnostic peaks are shown.

To identify what kinds of acetylated ether lipids occur in vivo in Halobacteria, we strived to analyze the lipids of two well‐characterized halobacterial species, *H. volcanii* H1424 and *H. salinarum* ATCC 700922. To this end, both strains were cultivated in the appropriate medium, cells were harvested, and TLEs were produced. Notably, like the hvYvoF protein solution, the lipid extracts were pink due to the carotenoids that are present in the halobacterial cells. The TLEs were then analyzed via reversed‐phase ultrahigh‐performance liquid chromatography (RP‐UHPLC) coupled with ESI‐MS. First, we screened the extract for acetylated mono‐ and dietherified glycerols. Figure 3 exemplarily shows the UHPLC elution profile and MS analysis for the *H. volcanii* extract, the data for *H. salinarum* are shown in Figure A6. The results are summarized in Table 1.

**Table 1:** Identified ether lipids in Halobacteria

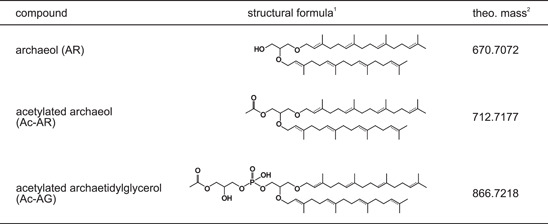

^1^Compounds were found with up to eight unsaturations, which is indicated by dashed double bonds.

^2^The theoretical (theo.) mass is calculated for the NH_4_^+^ adducts and is given for the saturated molecules only.

**Figure A7:**
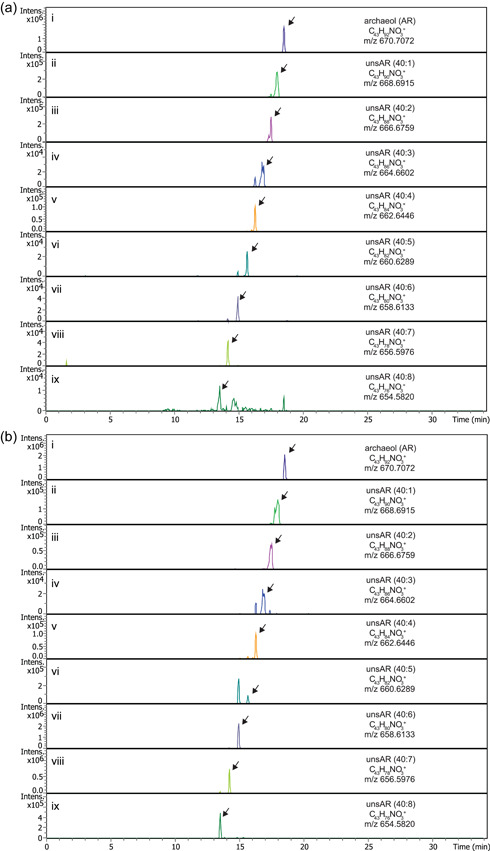
Detection of saturated and unsaturated ARs. Extracted ion chromatograms (0.01 Da width) of AR analogs with one to eight unsaturations (panel i–ix; uns, unsaturated; numbers in parentheses denote the number of carbons in both isoprenoid chains and the number of unsaturations in both chains) in (a) *Haloferax volcanii* and (b) *Halobacterium salinarum*. Black arrows point to the respective detected chromatographic peaks, minor peaks at earlier retention times represent isotopologues with two ^13^C atoms in unsAR with one more unsaturation or other compounds within the extracted mass window. AR, archaeol.

**Figure A8:**
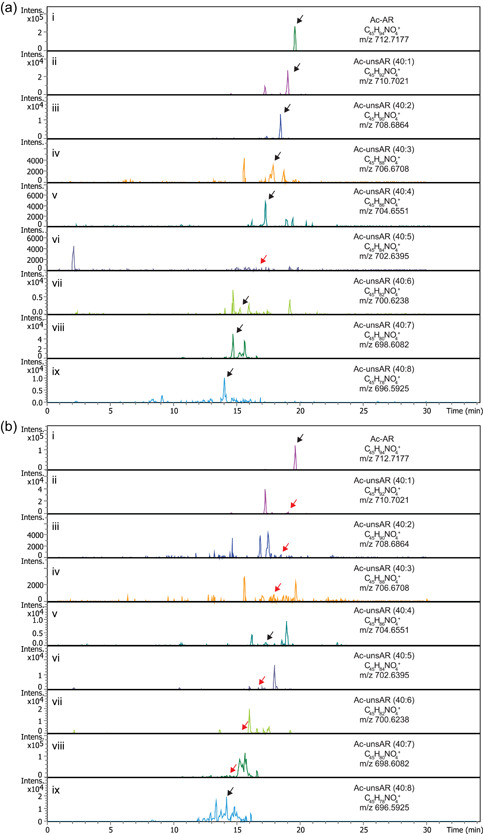
Detection of saturated and unsaturated Ac‐ARs. Extracted ion chromatograms (0.1 Da width) of Ac‐AR analogs with eight to eight unsaturations (panel i–ix; uns, unsaturated; numbers in parentheses denote the number of carbons in both isoprenoid chains and the number of unsaturations in both chains) in (a) *Haloferax volcanii* and (b) *Halobacterium salinarum*. Black arrows point to the respective detected chromatographic peaks, whereas red arrows indicate the expected retention time for compounds that could not be detected. Minor peaks at earlier retention times represent isotopologues with two ^13^C atoms in acetylated unsAR with one more unsaturation or other compounds within the extracted mass window. Ac‐AR, acetylated archaeol.

The mass spectral fragmentation patterns indicate that besides regular archaeol (AR, corresponding to saturated DGGG or 2,3‐diphytanyl‐*sn*‐glycerol; cf. Table 1), identified based on the characteristic dominant fragment of *m*/*z* 373.3676 from loss of one C20 isoprenoid chain as phytene (cf. Liu et al., 2012; Yoshinaga et al., 2011) and the less abundant fragment of *m*/*z* 355.3571 from loss of one C20 isoprenoid chain, including the OH‐group (Liu et al., 2012), acetylated archaeol (Ac‐AR) was present in the TLEs of both *H. volcanii* and *H. salinarum*. Ac‐AR was tentatively identified based on a characteristic fragment at *m*/*z* 397.3676 expected for a structure where one C20 isoprenoid chain including the OH‐group has been lost, and a fragment at *m*/*z* 117.0553 representing an acetylated glycerol derivative (see structural formulas in Figures 3b and A6B). Interestingly, the fragment corresponding to a loss of one C20 isoprenoid chain as phytene, that is, the dominant corresponding fragment of AR, is only barely visible for Ac‐AR (*m*/*z* 415.3781; peak not shown in the mass spectrum due to low abundance). The exact mechanism for the different fragmentation is currently unknown. In addition to their fully saturated molecules, ARs and Ac‐ARs were also detected with one to eight unsaturations (Figures A7 and A8) in both organisms. As expected for RP chromatography, the less polar Ac‐ARs elute approximately 1 min later compared to their nonacetylated counterparts, and additional unsaturations reduce the retention time relative to the saturated counterparts (cf. Wörmer et al., 2015). Because acetylated diether lipids are not commercially available, their mass spectrometric response factors cannot be determined and the proportion of Ac‐AR can only be estimated roughly, assuming that AR and Ac‐AR are detected in RP‐UHPLC‐ESI‐MS with the same mass spectrometric response. As derived from the peak heights (Figures 3B and A6), the content of fully saturated Ac‐AR would be approximately 10% compared to the amount of AR in the *H. volcanii* TLE, and approximately 5% in the *H. salinarum* TLE.

**Figure A9:**
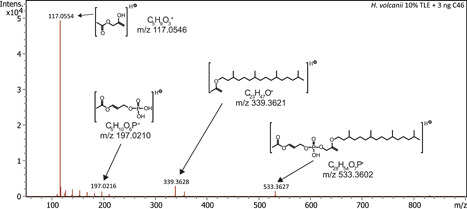
Mass spectrometry analysis of a *Haloferax volcanii* total lipid extract. Fragment mass spectrum of acetylated archaetidylglycerol (Ac‐AG). Tentative structures and elemental formulas of diagnostic fragment peaks are shown.

**Figure A10:**
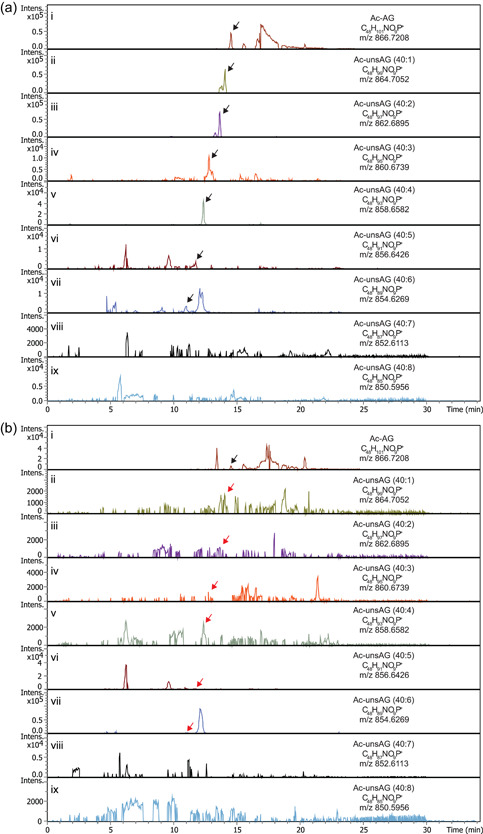
Detection of saturated and unsaturated acetylated archaetidylglycerol (Ac‐AG)s. Extracted ion chromatograms (0.1 Da width) of Ac‐AG analogs with 1–8 unsaturations (panel i–ix; uns, unsaturated; numbers in parentheses denote the number of carbons in both isoprenoid chains and the number of unsaturations in both chains) in *Haloferax volcanii* (a) and *Halobacterium salinarum* (b). Black arrows point to the respective detected chromatographic peaks, whereas red arrows show the expected retention time for compounds that could not be detected. Minor peaks at earlier retention times represent isotopologues with two ^13^C atoms in acetylated unsAR with one more unsaturation or other compounds within the extracted mass window.

**Figure A11:**
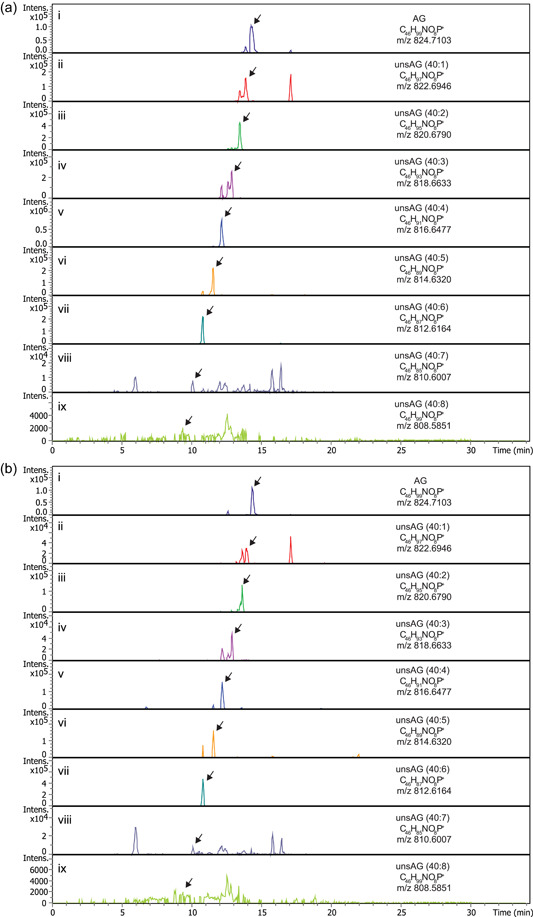
Detection of saturated and unsaturated archaetidylglycerols (AGs). Extracted ion chromatograms (0.1 Da width) of AG analogs with 1–8 unsaturations (panel i–ix; uns, unsaturated; numbers in parentheses denote the number of carbons in both isoprenoid chains and the number of unsaturations in both chains) in *Haloferax volcanii* (a) and *Halobacterium salinarum* (b). Black arrows point to the respective detected chromatographic peaks, whereas red arrows show the expected retention time for compounds that could not be detected. Minor peaks at earlier retention times represent isotopologues with two ^13^C atoms in acetylated unsAR with one more unsaturation or other compounds within the extracted mass window.

Interestingly, screening the UHPLC‐MS data for other compounds containing acetylated glycerol using the characteristic fragment at 117.05 Da (cf. Figure 3b) revealed additional acetylated lipids, namely archaetidylglycerols (Ac‐AG) (Figures A9 and A10, Table 1). Because archaetidylglycerol (AG) and AG methylphosphate (AG‐PCH_3_) are the main polar lipids of *H. volcanii* (Sprott et al., 2003), this finding seems reasonable. In *H. volcanii,* we could find almost all possible unsaturated structures of AG except for compounds with seven and eight double bonds (Figure A10A), while in *H. salinarum* we could only identify saturated Ac‐AG (Figure A10B). Unsaturated compounds were absent in this species. For AG, we found all possible unsaturated structures in both strains (Figure A11). As a rough quantitative estimation under the preconditions as stated above, the content of fully saturated Ac‐AG would be approximately 40% compared to the amount of AG in the *H. volcanii* TLE, as derived from the peak heights, and approximately 30% in the *H. salinarum* TLE.

Although we searched the RP‐UHPLC‐ESI‐MS results thoroughly, we did not find any signals of mono‐ or diacetylated monoether lipid derivatives similar to those found in *B. subtilis* (Figure 1a), even though hvYvoF can acetylate monoether lipids as suggested by our experimental data (Figure 2). In contrast to *B. subtilis*, Halobacteria possess a DGGGPS, which could rapidly process monoether to diether lipids and therefore be the reason for the absence of acetylated monoether lipids in the investigated strains under the given conditions.

Since headgroup modification and saturation of the isoprenoid chains normally happen after attachment of the cytidine diphosphate (CDP) to the lipid, there must be an additional synthesis branch for Ac‐AR, which we propose as presented in Figure 1b. Instead of those modifications that lead to the conventional archaeal ether lipids, we propose that DGGGP (=phosphorylated unsaturated AR [unsAR]) is dephosphorylated and subsequently acetylated resulting in Ac‐unsAR. Afterward, partial or complete saturation of the polyprenyl moiety may take place. Alternatively, halobacterial YvoF could be a promiscuous enzyme that accepts both saturated and unsaturated AR lipids as substrate. The existence of acetylated AG derivatives supports this and indicates that YvoF might also act on readily processed lipids with a phosphoglycerol head group. YvoF might primarily acetylate the most abundant ether lipids with free hydroxyl groups at glycerol in the cell without certain substrate specificity.

### 3.3 Substrate range of hvYvoF

The hypothesis of YvoF being a promiscuous enzyme agrees with previous findings that many *O*‐acetyltransferases show a broad substrate range. The closest relatives to YvoF, maltose acetyltransferase (MAT) and galactoside *O*‐acetyltransferase, are known to acetylate a variety of sugars with different efficiencies (Lo Leggio et al., 2003). Similarly, *B. subtilis* YvoF can acetylate maltose, and MAT can acetylate GGG in vitro (Linde et al., 2016).

**Figure A12:**
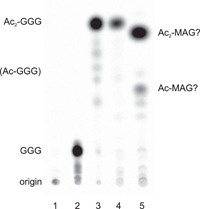
Substrate range of hvYvoF. GGG, monoacylglycerol, and controls were incubated with hvYvoF and acetyl‐CoA. Samples were analyzed on Silica 60 plates developed in ethyl acetate:hexane 1:1 (v:v) after extraction. (1) ^14^C‐acetyl‐CoA, no other substrate, no enzyme (negative control for extraction); (2) ^14^C‐GGG, no acetyl‐CoA, no enzyme (GGG control); (3) ^14^C‐GGG, unlabeled acetyl‐CoA (positive control for acetylation); (4) unlabeled GGG, ^14^C‐acetyl‐CoA; (5) monoacylglycerol, ^14^C‐acetyl‐CoA. For detailed reaction conditions, see Section 2. Note that for technical reasons, samples 2 and 3 contained 300 nCi of radioactivity, all other samples 40 nCi. To obtain spots of equal intensity, the sample volume applied to the TLC plate was reduced for samples 2 and 3. In each lane, 30 nCi were applied to the plate. The identities of the spots are labeled. ​​​​(Ac‐GGG), the position of Ac‐GGG, which was only produced in minor amounts in lanes 3 and 4 and is therefore not visible;  ?, putative identification, the identity of the spot has not been confirmed within this study or previously by MS; GGG, geranylgeranylglycerol; MAG, monoacylglycerol; hvYvoF, *Haloferax volcanii YvoF*; TLC, thin‐layer chromatography.

To further support this hypothesis, we qualitatively tested whether hvYvoF can acetylate other lipid substrates than GGG. To this end, we incubated hvYvoF with [1‐^14^C]‐labeled acetyl‐CoA and monoacylglycerol (Figure A12). The obtained products (lane 5) show a clearly different running behavior in chromatography than the control samples with GGG (lanes 3 and 4) and indicate that YvoF can also acetylate ester lipids. Diacylglycerol turned out to be not soluble under the given assay conditions, making it very likely that AR would not be soluble, too. In vitro acetylation experiments with AR and AG would prove that it is YvoF that produces the Ac‐AR and Ac‐AG we found in vivo, but because of the solubility problems and because AR and AG are difficult to obtain, we refrained from such experiments. Nevertheless, together with previous data (Linde et al., 2016), our novel results strongly suggest that YvoF can acetylate a large number of glycerol derivatives, most likely also AR and AG. Previous results indicate that YvoF is a membrane‐associated protein (Linde et al., 2016), making it likely that lipids are its primary substrate.

Another direct evidence would be the creation of a *yvoF* knockout strain in a halobacterial species, which is a laborious task, with no certainty that such a knockout strain would be viable and creatable at all. As an alternative to this, we have analyzed the content of Ac‐AR and Ac‐AG in an *H. volcanii* strain that overexpresses *yvoF*, namely the strain we used for the production of the hvYvoF protein, following the hypothesis that this strain might have elevated levels of acetylated lipids. However, MS analysis of TLEs from this strain revealed no significant difference in weight. One reason for this observation might be that acetylation is regulated by additional means other than the expression level of YvoF.

## 4 CONCLUSION

Our results demonstrate that halobacterial YvoF exhibits GGG acetyltransferase activity in vitro, just like its homolog from *B. subtilis* (Linde et al., 2016). This matches our mass spectrometric identification of Ac‐AR, Ac‐AG, and various unsaturated derivatives thereof in *H. volcanii* and *H. salinarum*. We suggest that YvoF is responsible for acetylating these lipids in both species (the sequence identity of their YvoF protein sequences is 67%), and that acetylated ether lipids might also occur in many other Halobacteria that possess a YvoF homolog.

As outlined in our proposed pathway (Figure 1b), our findings suggest that YvoF catalyzes acetylation of diether lipids in Halobacteria after the second isoprenoid chain has been added by DGGGPS because we were unable to detect any acetylated monoether lipids. DGGG might be much preferred over GGG by YvoF in vivo*.* Experiments with *B. subtilis* YvoF indicated that it is a membrane‐associated protein (Linde et al., 2016), which makes it likely that it acts on lipids that are located there. Most likely, acetylation happens in parallel to the reduction of the isoprenoid moieties by DGGGPR and the attachment of glycerol as a polar head group, as we found a series of different partially unsaturated Ac‐AR and Ac‐AG derivatives. We suppose that the acetyl group might serve alone as a small headgroup that reduces the polarity of the glycerol core of AR or serves to modify the polarity of the glycerol headgroup in the case of AG. It remains elusive how acetylation is in balance with the attachment of other head groups which cannot be acetylated.

According to a previous study (Sprott et al., 2003), the main polar membrane lipids of *H. volcanii* are archaetidylglycerol methylphosphate (AG‐PCH_3_; 44% of total lipid) and AG (35%), followed by sulfated glycolipids (14%), archaeal cardiolipin (5%), and archaetidic acid (2%). As a rough estimation for *H. volcanii*, the content of fully saturated Ac‐AR was about 10% of that of the detected amount of AR, and the amount of Ac‐AG was 40% of that of AG. Based on these assumptions, the total content of acetylated ether lipids in a low percentage range seems reasonable and might be similar in *H. salinarum.*

Since YvoF does not occur in other Archaea than Halobacteria, it probably migrated from the Bacillales into the archaeal domain of life or vice versa by horizontal gene transfer, and due to the significant amounts of acetylated lipids we detected, they probably fulfill a distinct function. The biological role of the acetylated monoether lipids that have been found in Bacillales is still unclear, and the discovery of acetylated diether lipids in the archaeal domain of life makes it even more interesting to study them in more detail.

## AUTHOR CONTRIBUTIONS

**Cosimo Kropp**: Conceptualization (equal); data curation (equal); formal analysis (equal); investigation (equal); writing—original draft (equal); writing—review and editing (equal). **Julius Lipp**: Data curation (equal); formal analysis (equal); investigation (equal); writing—original draft (equal); writing—review and editing (equal). **Anna Lena Schmidt**: Investigation (supporting); writing—review and editing (supporting). **Christina Seisenberger**: Investigation (equal); writing—review and editing (supporting). **Mona Linde**: Investigation (supporting); supervision (equal); writing—review and editing (supporting). **Kai‐Uwe Hinrichs**: Resources (equal); writing—review and editing (equal). **Patrick Babinger**: Conceptualization (equal); data curation (equal); formal analysis (equal); project administration (lead); supervision (lead); writing—original draft (equal); writing—review and editing (equal).

## CONFLICT OF INTEREST

None declared.

### 1 ETHICS STATEMENT

None required.

## Data Availability

All the data are available in this published article.

## 1

(Table A1, Figures A1, A2, A3, A4, A5, A6, A7, A8, A9, A10, A11, A12).

**Table A1:** Primers used for amplification and cloning of hv*yvoF* into pTA1228 vector.

Primer	Sequence (5′ ⟶ 3′)^a^
hv*yvoF*_*Nde*I_fwd	CTC**CATATG**ACCGACGACGATGCCGCACCG
hv*yvoF*_*Xho*I_rev	TGCG**CTCGAG**CGCTCCGTCAGTCTCGTCGC

^a^ Restriction sites are printed in bold letters.
